# RETRACTION: The Effects of Intravenous Lidocaine on Wound Pain and Gastrointestinal Function Recovery After Laparoscopic Colorectal Surgery

**DOI:** 10.1111/iwj.70642

**Published:** 2025-04-23

## Abstract

**RETRACTION:** WeiS. , Yu‐hanZ. , Wei‐weiJ. , and HaiY. , “The Effects of Intravenous Lidocaine on Wound Pain and Gastrointestinal Function Recovery After Laparoscopic Colorectal Surgery,” International Wound Journal 17, no. 2 (2020): 351–362, 10.1111/iwj.13279.31837112 PMC7949458

The above article, published online on 13 December 2019, in Wiley Online Library (http://onlinelibrary.wiley.com/), has been retracted by agreement between the journal Editor in Chief, Professor Keith Harding; and John Wiley & Sons Ltd. Following an investigation by the publisher, all parties have concluded that this article was accepted solely on the basis of a compromised peer review process. The editors have therefore decided to retract the article. The authors did not respond to our notice regarding the retraction.

**RETRACTION:** S. Wei , Z. Yu‐han , J. Wei‐wei , and Y. Hai , “The Effects of Intravenous Lidocaine on Wound Pain and Gastrointestinal Function Recovery After Laparoscopic Colorectal Surgery,” International Wound Journal 17, no. 2 (2020): 351–362, 10.1111/iwj.13279.31837112 PMC7949458

The above article, published online on 13 December 2019, in Wiley Online Library (http://onlinelibrary.wiley.com/), has been retracted by agreement between the journal Editor in Chief, Professor Keith Harding; and John Wiley & Sons Ltd. Following an investigation by the publisher, all parties have concluded that this article was accepted solely on the basis of a compromised peer review process. The editors have therefore decided to retract the article. The authors did not respond to our notice regarding the retraction.

